# Combining Membrane Potential Imaging with l-Glutamate or GABA Photorelease

**DOI:** 10.1371/journal.pone.0024911

**Published:** 2011-10-11

**Authors:** Kaspar E. Vogt, Stephan Gerharz, Jeremy Graham, Marco Canepari

**Affiliations:** 1 Division of Pharmacology and Neurobiology, Biozentrum – University of Basel, Basel, Switzerland; 2 CAIRN Research Ltd., Faversham, United Kingdom; 3 Inserm, U836, Team 3, BP 170, Grenoble, France; 4 Université Joseph Fourier, Institut des Neurosciences, BP 170, Grenoble, France; Institut National de la Santé et de la Recherche Médicale, France

## Abstract

Combining membrane potential imaging using voltage sensitive dyes with photolysis of l-glutamate or GABA allows the monitoring of electrical activity elicited by the neurotransmitter at different sub-cellular sites. Here we describe a simple system and some basic experimental protocols to achieve these measurements. We show how to apply the neurotransmitter and how to vary the dimension of the area of photolysis. We assess the localisation of photolysis and of the recorded membrane potential changes by depolarising the dendrites of cerebellar Purkinje neurons with l-glutamate photorelease using different experimental protocols. We further show in the apical dendrites of CA1 hippocampal pyramidal neurons how l-glutamate photorelease can be used to calibrate fluorescence changes from voltage sensitive dyes in terms of membrane potential changes (in mV) and how GABA photorelease can be used to investigate the phenomenon of shunting inhibition. We also show how GABA photorelease can be used to measure chloride-mediated changes of membrane potential under physiological conditions originating from different regions of a neuron, providing important information on the local intracellular chloride concentrations. The method and the proof of principle reported here open the gateway to a variety of important applications where the advantages of this approach are necessary.

## Introduction

In physiological research, optical techniques offer the possibility to stimulate and record from multiple sub-cellular sites, potentially with high spatial resolution. The advantages of optical methods are greater when optical stimulation is combined with optical recording. Chemical stimulation can be delivered optically by photorelease of a molecule from a caged compound [1]. Because most available caged compounds do not photorelease above a certain wavelength, uncaging stimulation can be optimally combined with fluorescence recording if the light used to excite the fluorescent molecule is inert to the caged compound. A notable example is the recording of Ca^2+^ signals associated with photorelease of inositol triphosphate, obtained initially at broad spatial resolution [2], [3] and later with a resolution in the order of a micron [4]. Similarly, Ca^2+^ signals mediated by synaptic glutamate receptors were recorded from neuronal dendrites after large-field l-glutamate photorelease [5], [6], or recorded from dendritic spines after two-photon l-glutamate photorelease [7].

Whereas simultaneous Ca^2+^ imaging and photorelease provides information on the chemical activity produced by a particular molecule, membrane potential imaging using voltage sensitive dyes [8] can be combined with uncaging to investigate how the photorelease of a molecule changes the membrane potential in different regions of a cell. In this study, we describe procedures to obtain simultaneous single-cell membrane potential optical measurements [9], [10] and large-field photorelease. We use the caged compounds 4-Methoxy-7-nitroindolinyl-caged-l-glutamate (MNI-glutamate) [11]–[13] and 1-(4-Aminobutanoyl)-4-[1,3-*bis*(dihydroxyphosphoryloxy)propan-2-yloxy]-7-nitroindoline (DPNI-GABA) [14], [15] to obtain fast photorelease of either l-glutamate or GABA in sub-cellular regions of variable size. We assess the method by recording optically from the dendrites of cerebellar Purkinje neurons (PNs) the depolarisation elicited by l-glutamate photorelease and we present a series of important applications using this approach.

## Methods

### Preparations, solutions, electrophysiology and analysis

Experiments were conducted in Basel, Switzerland, and were permitted by the Veterinary office of the Canton of Basel-Stadt (permit number 219). Mice used in these experiments were from licensed and controlled breeding facilities, either at the University of Basel or from licensed commercial vendors (Harlan). The local mouse facility, supervised by the veterinary authorities and staffed by licensed professionals, provided for the transfer of the animals to the laboratory on the same day as the experiments. Experiments on organs ex vivo (such as the ones performed here) were reported to the veterinary office. The euthanasia procedure, approved by the veterinary authorities, consisted of deep isoflurane anesthesia, tested by hind paw reflex and observation, followed by rapid decapitation and cooling of the brain. The permit number 219 covers these experiments with post-euthanasia brain removal classified among those with degree of severity 0 and not requiring formal approval by an ethics committee.

Experiments were performed in 250 µm thick cerebellar sagittal slices or hippocampal transversal slices from 27–35 days old mice (C57BL/6) prepared as previously described [10], [16], [17]. The extracellular solution used for recordings contained (mM): 125 NaCl, 26 NaHCO_3_, 20 glucose, 3 KCl, 1 NaH_2_PO_4_, 2 CaCl_2_ and 1 MgCl_2_, bubbled with 95% O_2_ and 5% CO_2_. The intracellullar solution used for patch clamp recordings, i.e. for single-cell dye staining and for concomitant somatic membrane potential measurements, contained (mM): 125 KMeSO_4_, 5 KCl, 8 MgSO_4_, 5 Na_2_-ATP, 0.3 Tris-GTP, 12 Tris-Phosphocreatine, 20 HEPES, adjusted to pH 7.35 with KOH. Tetrodotoxin (TTX) citrate and 2,3-Dioxo-6-nitro-1,2,3,4-tetrahydrobenzo[*f*]quinoxaline-7-sulfonamide (NBQX) disodium salt were purchased from Tocris (Bristol, UK) and dissolved in water. Cyclothiazide (CTZ) was purchased from Tocris and pre-dissolved in DMSO at 100 mM concentration. MNI-glutamate and DPNI-GABA were purchased from Tocris and dissolved in extracellular solution. Patch-clamp recordings were made using a Multiclamp amplifier 700A (Molecular Devices, Sunnyvale, CA). Somatic membrane potentials were filtered at 4 kHz and acquired at 16 kHz. In patch recordings, the absolute value of the membrane potential was estimated after correcting for the junction potential of −11.0 mV calculated using JPCalc software [18].

Individual neurons in slices were loaded with JPW1114 (0.2–0.5 mg/mL) and intracellular staining was accomplished as previously described in detail [16]. Temperature during measurements was 32°–34°. Recordings were analysed using Matlab (The MathWorks Inc., Natick, MA). Non-calibrated optical signals were reported as fractional changes in fluorescence (ΔF/F). Anatomical reconstruction of neurons was made from two-photon images as previously described [17].

### Apparatus and experiments design

**Figure 1:**
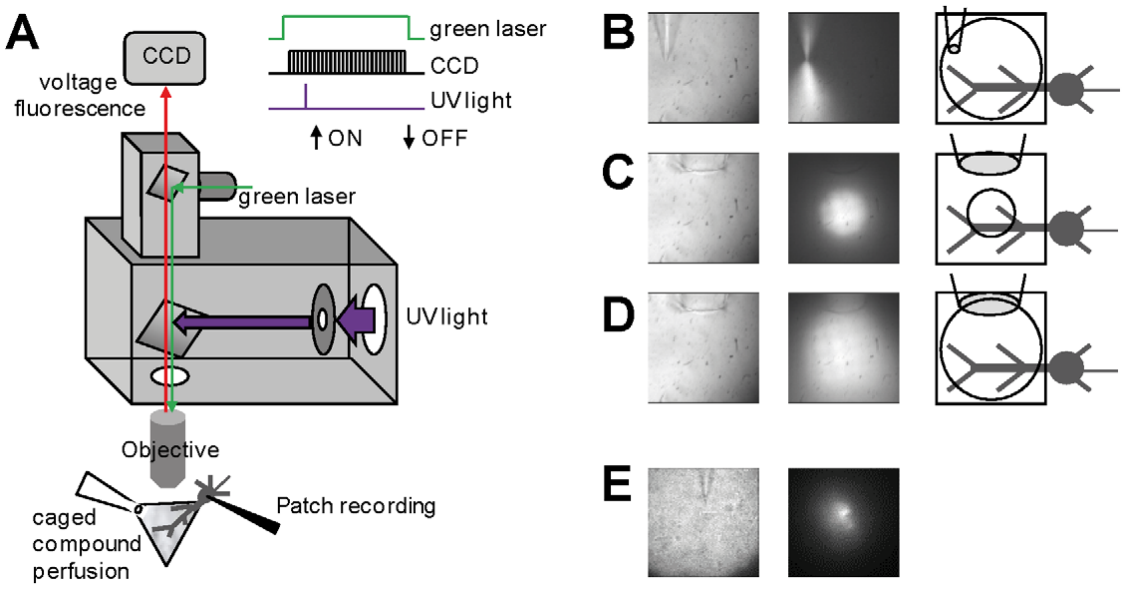
Imaging and uncaging apparatus. **A.** Schematic drawing of the apparatus for combined voltage imaging and l-glutamate and GABA photo-release; 365 nm OptoLED (CAIRN) illumination via the epifluorescence port of the microscope reflected by a 506 nm long-pass dichroic mirror; 532 nm laser illumination via the top of the microscope is reflected by a 570 nm long-pass dichroic mirror; the timing of the dual illumination and of the image acquisition is shown on the right (duration of the UV pulse: 0.1–1 ms); the caged compound is locally applied through a glass pipette; the voltage fluorescence is demagnified, filtered by a 610 nm long-pass filter and acquired by a NeuroCCD camera (RedShirtImaging); the whole-field of the camera (80×80 pixels) corresponds to 160 µm×160 µm. **B–D.** Configurations of local perfusion and UV-illumination; (left)- transmitted light images showing the size of the application electrode; (middle)- fluorescence images with the electrode delivering a fluorescent solution excited by the UV pathway; (right)- schematic of the uncaging mode showing the size of the application electrode and the size of the illumination spot; local photolysis is obtained either by applying the caged compound with a patch pipette and illuminating the whole field (panel B) or by applying the caged compound with a large pipette and reducing the illumination with a field-stop (panel C); photolysis over the whole field of view is obtained using the configuration in panel D. **E.** Profile of local perfusion with a patch pipette on a brain slice; (left)- transmitted light image; (right)- fluorescence image.

The system designed for simultaneous voltage imaging with L-glutamate or GABA photorelease is shown in Figure 1A. This system is based on a BX51 Olympus microscope modified to achieve simultaneous wide-field illumination using two light sources [19]. UV illumination used for photolysis was provided by a 365 nm LED controlled by an OptoLED (CAIRN Research Ltd., Faversham, UK). The LED was mounted on the epifluorescence port of the microscope and switched on and off by a TTL pulse generated with a Master-8 (A.M.P.I., Jerusalem, Israel). UV light was directed by a 506 nm long-pass dichroic mirror to a Nikon 60×/1.0 NA objective. The maximum power of the LED was limited by the protection circuit of the OptoLED and corresponded to ∼50 mW at the objective during continuous illumination. The maximal intensity, however, was 2–3 times higher for illuminations lasting 0.1–1 ms used for photolysis. To excite voltage fluorescence, a custom-made unit was designed permitting wide-field illumination of the preparation from above the objective using a 300 mW 532 nm solid state laser (model MLL532; CNI, Changchun, China). The light of the laser was directed to the objective using a 570 nm long-pass dichroic mirror. The image of the preparation was demagnified by ∼0.19×, long-pass filtered at 610 nm and acquired by a NeuroCCD-SM camera (RedShirtImaging LLC, Decatur, GA). The acquired field corresponded to ∼160 µm×160 µm. The laser was kept constantly on and the illumination was controlled by a TTL-triggered shutter (Vincent Associates, Rochester, NY). The output channels of the A/D board of the NeuroCCD-SM were used to control the shutter and to trigger the Master-8, used to control the UV illumination.

Images were acquired at 2 kHz using 25% of the full light of the laser (300 mW). This light intensity was selected to make optimal use of the dynamic range of the CCD sensor (well depth 215,000 electrons). The exposure time of a single trial was 50–150 ms except in the photolysis trials used for fluorescence change calibration. No photodynamic damage was observed after 20 exposure periods separated by 30–60 s confirming the results of previously reported tests [17]. Data reported in the figures were either from single trials or from averages of 4–9 trials as specified in the figure legends. The amount of neurotransmitter photoreleased was increased or decreased either by changing the duration of the UV pulse or by changing the intensity of the UV light. The UV pulse was apparent in the recorded light in the exposed area. The consequent artefact was not erased from the traces reported in the figures as so to indicate the timing of the photolysis.

### Caged compound application and photolysis area

Bath application of caged compounds suffers from two problems. Firstly, if water-dipping objectives with working distance >1 mm are used, the light absorption between the objective and the preparation significantly limits the efficiency of uncaging at the focal plane. This problem can be overcome by using longer wavelengths for uncaging, for example 405 nm for MNI-glutamate and DPNI-GABA [20]. Secondly, continuous bath perfusion of the precursor at millimolar concentrations, (necessary to mimic physiological release), requires large amounts of the caged compound and consequently very high costs for the experiments. To reduce the amount of caged compounds and to minimise the depth of the absorbing medium we delivered the precursor using local perfusion. This approach, however, prevents a precise determination of the concentration of caged compound at the site of release and therefore limits the precision of the calibration. Two techniques were utilised for local perfusion. Using the first technique we filled a patch electrode of ∼2 MΩ resistance, (Figure 1B, left), with the external solution containing 3 mM of either MNI-glutamate or DPNI-GABA. We delivered the solution to the surface of the slice by applying a pressure of ∼10 mbar. The beam of delivery of a fluorescent medium (Figure 1B, centre) gives a qualitative indication of the spatial distribution of the perfused medium. With this type of local perfusion, we illuminated the whole field of the objective (Figure 1B, right). As an alternative method we delivered the external solution containing 1 mM of the caged compound through gravity perfusion using a glass electrode with a tip of 50–100 µm at a speed of ∼0.05 mL/min (Figure 1C–D, left). As shown by the fluorescence in Figure 1C (centre), the area of photolysis could be restricted using the field-stop of the microscope (Figure 1C, right). Alternatively, the entire visualised area could be exposed to UV light (Figure 1D, centre) by using wide-field illumination (Figure 1D, right). The perfusion profile using a patch pipette (Figure 1E, left) is shown in the right panel of Figure 1E.

## Results

### Assessment of photolysis by using L-glutamate photorelease in PNs

**Figure 2:**
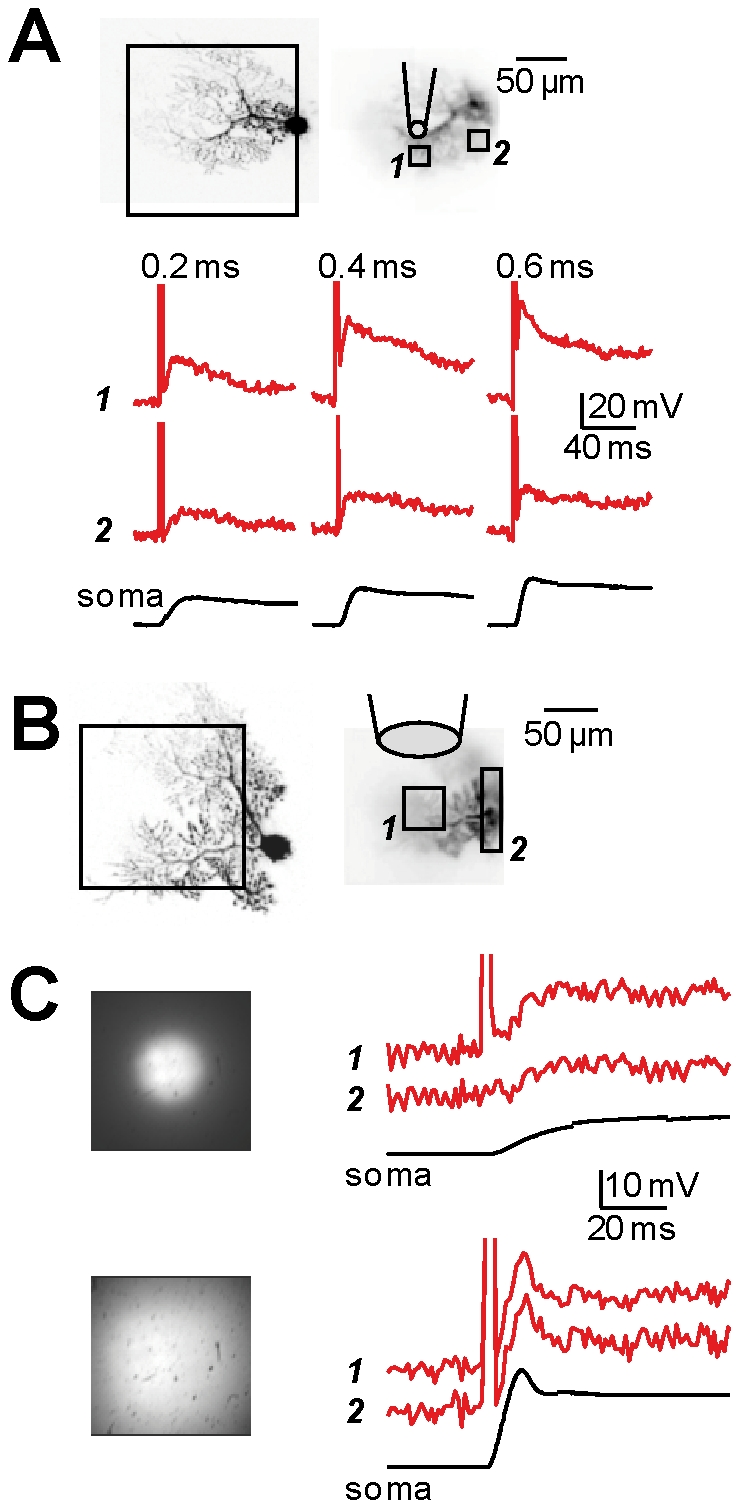
Dendritic excitation of PNs by l-glutamate photolysis using different configurations. **A.** (Top)-Image of a PN; the dendritic area in the recording position is outlined and two regions of interest (ROIs) (∼20 µm×20 µm) are shown on the right; caged-glutamate is applied locally using a patch pipette (Figure 1B). (Bottom)- ΔV_m_ optical signals from ROIs *1* and *2* (red traces) and somatic recordings (black traces) associated with l-glutamate photorelease with different pulse durations. **B.** From another PN, the dendritic area in recording position is outlined and two ROIs (∼40 µm×40 µm and 20 µm×80 µm) are shown on the right; caged-glutamate is applied over the whole dendrite using a large pipette. **C.** (Top)- ΔV_m_ optical signals from ROIs *1* and *2* in B (red traces) and somatic recordings (black traces) associated with l-glutamate photorelease with 1 ms pulse durations; the field of illumination is reduced by the field-stop of the microscope (Figure 1C); notice the absence of artefact caused by UV illumination in ROI *2*. (Bottom)- ΔV_m_ optical signals from ROIs *1* and *2* in B (red traces) and somatic recordings (black traces) associated with wide-field l-glutamate photorelease (configuration shown in Figure 1D) with 1 ms pulse durations. Experiments were performed in the presence of 1 µM TTX. All traces are averages of 4 trials.

To assess our system we performed experiments of simultaneous voltage imaging and l-glutamate photorelease in 6 PNs. The general condition, in single-cell voltage imaging experiments, is that fractional changes cannot be calibrated in terms of membrane potential changes (in mV). However, in PNs, prolonged hyperpolarising somatic pulses spread in the dendrites with minimal attenuation [21] and can be used to calibrate fluorescence signals to estimate the change in membrane potential associated with any stimulation [10]. We used this property of PNs to compare somatic depolarisation with the depolarisation recorded in different areas of the dendritic tree. We performed these experiments in the presence of 1 µM TTX to block somatic Na^+^ spikes. In three cells, we applied MNI-glutamate using a patch pipette (Figure 1B). In the representative example of Figure 2A, we compared the membrane potential changes following l-glutamate photorelease events of 0.2 ms, 0.4 ms and 0.6 ms duration in a region of interest (ROI) near the perfusion pipette (ROI *1*) and in another ROI (*2*) of the same size located ∼60 µm away from ROI *1*. For all durations of the UV pulses, the depolarisation in ROI *1* was more than double the depolarisation observed in ROI *2*, in the soma and in all areas of the dendrite far from the perfusion pipette (data not shown). In an additional 3 cells, we applied the MNI-glutamate locally using the configuration shown in Figure 1C–D. In the representative example shown in Figure 2B, we analysed the membrane potential changes following one l-glutamate photorelease event of 1 ms, either using the configuration of reduced-field of UV illumination (Figure 1C) or using the configuration of wide-field UV illumination (Figure 1D). In this case, we compared the signals in ROI (*1*) centred in the reduced-field of illumination with ROI (*2*) outside that field (Figure 2B). The absence of optical artefact generated by the UV pulse was used to verify that the particular ROI was outside the field of UV illumination (Figure 2C, top traces). The depolarisation was larger in ROI *1* when l-glutamate was released by reduced-field illumination (Figure 2C, top traces), but it was uniform in the case of wide-field illumination (Figure 2C, bottom traces). Altogether, data in Figure 2 show that non-uniform or uniform membrane potential changes can be produced and recorded from individual neurons by using the different configurations illustrated in Figure 1B–D. Using UV LED illumination from the epifluorescence port of the microscope, the best spatial resolution of photolysis is of the order of tens of microns. A UV laser can be used to obtain a resolution in the order of a micrometer [4], [20], [22], necessary to mimic activation of individual synapses. Wide-field or reduced-field photolysis obtained with UV LED illumination can be used for other applications where neurotransmitter release with the resolution of one or a few microns is not necessary. Some of these applications are described below.

### Use of L-glutamate photorelease to calibrate membrane potential optical signals

As mentioned in the previous paragraph, fractional changes of fluorescence from voltage sensitive dyes cannot be calibrated per se in terms of membrane potential change on an absolute scale. This limitation prevents the comparison of electrical activity from different sites. In the case of PNs or of olfactory bulb mitral cells [23], a calibration is possible because a calibrating electrical signal of known amplitude is available at all locations. The possibility to achieve uniform depolarisations over the entire dendritic tree using L-glutamate photolysis may extend the possibility to calibrate fluorescence signals to other cell types. This calibration procedure is based on the principle that if the ionotropic glutamate receptor becomes the dominant conductance in a particular neuronal compartment, its reversal potential will determine the membrane potential of the compartment. Thus, if dominance of glutamate receptor conductance is obtained by L-glutamate uncaging, the resulting membrane potential change in different compartments of the neuron will be the same and can be used to calibrate voltage-sensitive dye imaging data.

**Figure 3:**
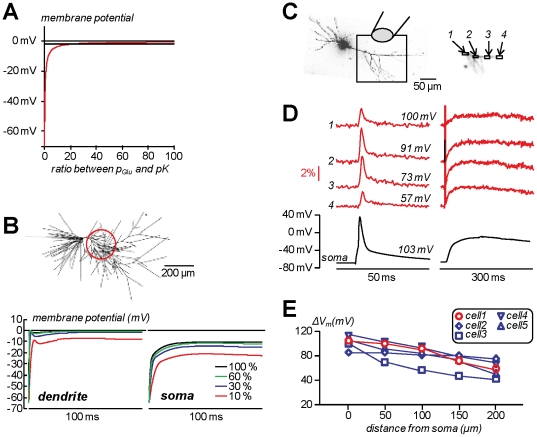
Calibration of fractional changes of fluorescence in terms of membrane potential using wide-field l-glutamate photorelease. **A.** From the Goldman Equation (1), plot of calculated membrane potential against the ratio between the non-selective cation conductance (*p_Glu_*) and the K^+^ conductance (*pK*). **B.** (Top)- Model CA1 hippocampal pyramidal neuron from [28]; the red circle indicates a putative area of imaging and photolysis. (Bottom)- NEURON simulation of the time course of the membrane potential following activation of randomly positioned glutamate synapses in the photolysis area indicated above using the model adapted from [28]; parameters were from [28] with the exception of the absence of voltage-gated Na^+^ channels and with 200 ms decay time of AMPA receptors (instead of 20 ms); different colours indicate different percentage of activated synapses from the number (1700) estimated in [27]. **C.** Image of a CA1 hippocampal pyramidal neuron; the dendritic area in the recording position is outlined and four ROIs (∼16 µm×8 µm) are shown on the right; caged-glutamate is applied over the visualised area of the dendrite and wide-field illumination was used for photolysis (configuration of Fig. 1C). **D.** (Left)- Membrane potential optical signals from ROIs *1*–*4* (red traces) and somatic recording (black trace) associated with one back-propagating action potential elicited by somatic current injection. (Right)- After addition of 1 µM TTX and 50 µM CTZ, membrane potential optical signals from ROIs *1*–*4* (red traces) and somatic recording (black trace) associated with 1 ms l-glutamate photo-release. The peak of the somatic action potentials is indicated over its trace; the sizes of the estimated peak spikes in the ROIs are indicated over their respective traces; all traces are from single trials. **E.** Plot of the estimated peak of the spike against the distance from the soma in the cell shown in A (red symbols) and in four other cells (blue symbols).

We quantitatively address whether this situation realistically occurs in CA1 hippocampal pyramidal neurons in the case of prolonged changes of conductance where the contribution of voltage-gated conductance is negligible compared to leak conductance [24], [25]. Under this condition, the membrane potential due to the activation of non-selective cation conductance (*p_Glu_*) and to K^+^ conductance (*pK*) is given by the Goldman equation:
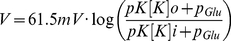
(1)
Figure 3A shows the plot of the membrane potential calculated from Equation 1 against the ratio between *p_Glu_* and *pK*. At ratios >20, the membrane potential approaches the reversal potential of the non-selective cation conductance to within less than 2 mV. The input resistance of mouse CA1 pyramidal neurons has been estimated at between 65 MΩ and 200 MΩ [26]. Thus, over the surface of 20·10^3^ µm^2^, (realistic for a mouse CA1 pyramidal neuron), the K^+^ conductance density is <0.77 pS/µm^2^ [26]. AMPA current density in CA1 pyramidal neurons has been measured to be 20 pA/µm^2^ (secondary dendrites) and 40 pA/µm^2^ (tertiary dendrites), which translates into conductance densities (60 mV driving force) of 330 pS/µm^2^ (secondary dendrites) and 660 pS/µm^2^ (tertiary dendrites) [27]. Assuming an even leak conductance distribution, *p_Glu_*/*pK* will be >430 at secondary dendrites and >860 at tertiary dendrites. Thus, the condition for reaching the reversal potential of glutamate receptors can be obtained by activation of 5% of the available AMPA receptors.

To confirm that a uniform membrane potential over the illuminated area is obtained by l-glutamate photorelease, we ran a *NEURON* computer simulation using a published model of CA1 hippocampal pyramidal neuron [28]. In this model, resting membrane potential is uniform over the whole cell, an approximation consistent with the evidence that the differences in resting membrane potential along the apical dendrites of these neurons are <2 mV [24]. From the existing parameters of this model, we mimicked the presence of TTX by eliminating voltage-gated sodium conductance and we also mimicked a CTZ-induced block of AMPA receptor desensitization by changing the decay time of AMPA receptors from 20 ms to 200 ms. The schematic of the model neuron is shown in Figure 3B (top). According to the mean synapse density reported in the literature [27], we estimated a number of 1700 glutamate synapses on the putative area of photolysis indicated by the red circle of 200 µm diameter. We then simulated the effect of glutamate uncaging by activation of randomly distributed synapses in the photolysis area. In Figure 3B (bottom) the simulated membrane potential change in a dendritic region of the photolysis area (left) and in the soma (right) are reported. The traces correspond to activation of 10%, 30%, 60% and 100%, mimicking the activation of progressively higher numbers of receptors. As shown in the plots, the dendritic membrane potential approaches 0 mV when 30% of the total number of synapses are activated, whereas the somatic membrane potential remains slightly negative. The idea of a calibration protocol based on l-glutamate uncaging relies on producing a saturating depolarisation in the visualised area. The fractional change of fluorescence will then correspond to the resting membrane potential in each visualised site where photolysis occurs.

We then applied the calibration procedure to measure the progressive attenuation of the back-propagating action potential in CA1 hippocampal pyramidal neurons [29]. In the cell of Figure 3C, one action potential was evoked by somatic current injection. The fractional changes of fluorescence associated with the back-propagation of the action potential from four ROIs on the apical dendrites are shown in Figure 3D (left traces). The amplitude of the signal is variable and could not be directly correlated with the absolute change of membrane potential. Thus, we added 1 µM TTX, to block action potentials, and 50 µM CTZ, to prolong depolarisation by blocking AMPA receptor desensitisation, to produce the conditions of the simulation shown in Figure 3B. Under these conditions, we photoreleased L-glutamate over the whole field of view (Figure 1D). The recording was performed starting from the resting membrane potential of the cell. The size of the fluorescence change at each site and the absolute somatic depolarisation elicited by uncaging increased with the amount of released L-glutamate, i.e. with the exposure time of the UV light, eventually reaching saturation. According to the simulation reported in Figure 3B, the saturating membrane potential shown in Figure 3D (right traces) will correspond to the reversal potential of glutamate receptors. Using this information, we converted the amplitude of the back-propagating action potential optical signals into the change in membrane potential. In 5 cells tested, the amplitude of the back-propagating action potential declined with the distance from the soma (Figure 3E).

The proposed method of calibration based on L-glutamate photorelease can only be applied to cell types where the principle of dominance of glutamate conductance over leak conductance is valid. In general, if the resting membrane potential is not uniform over the illuminated part of the dendrite, the error in the calibration will be given by the ratio of the difference of resting membrane potentials and the voltage transient elicited by L-glutamate photorelease.

### Use of GABA photorelease to investigate Cl^−^ mediated potentials under physiological conditions

**Figure 4:**
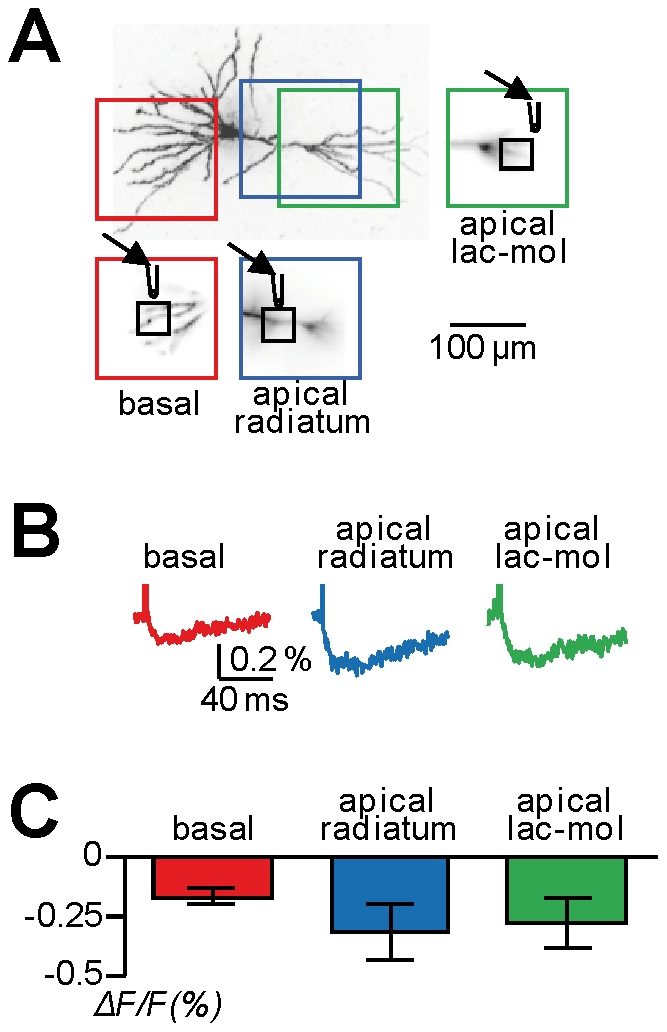
Polarity of dendritic signals in response to wide-field GABA photorelease in basal dendrites, proximal apical dendrites and distal apical dendrites in CA1 hippocampal pyramidal neurons. **A.** Image of a CA1 hippocampal pyramidal neuron; three dendritic areas in recording position are outlined in red (basal dendrites), blue (apical dendrite in the stratum radiatum) and green (apical dendrite in the stratum lacunosum-moleculare); the corresponding fluorescence images are reported on the bottom and on the right each with a ROI (∼20 µm×20 µm) and the position of local perfusion (configuration of Figure 1B) indicated. **B.** From cell in A, membrane potential optical signals associated with GABA photorelease by UV applications of 0.6 ms without patch recording from the basal dendrites (red trace), from the proximal apical dendrite (apical radiatum, blue trace) and from the distal apical dendrite (apical lac-mol, green trace); all traces are from averages of 9 trials. **C.** Mean ± SD (N = 5 cells) of the dendritic membrane potential optical signals associated with GABA photorelease by UV applications of 0.6 ms without patch recording from the basal dendrites (red column), from the proximal apical dendrite (apical radiatum, blue column) and from the distal apical dendrite (apical lac-mol, green column).

**Figure 5:**
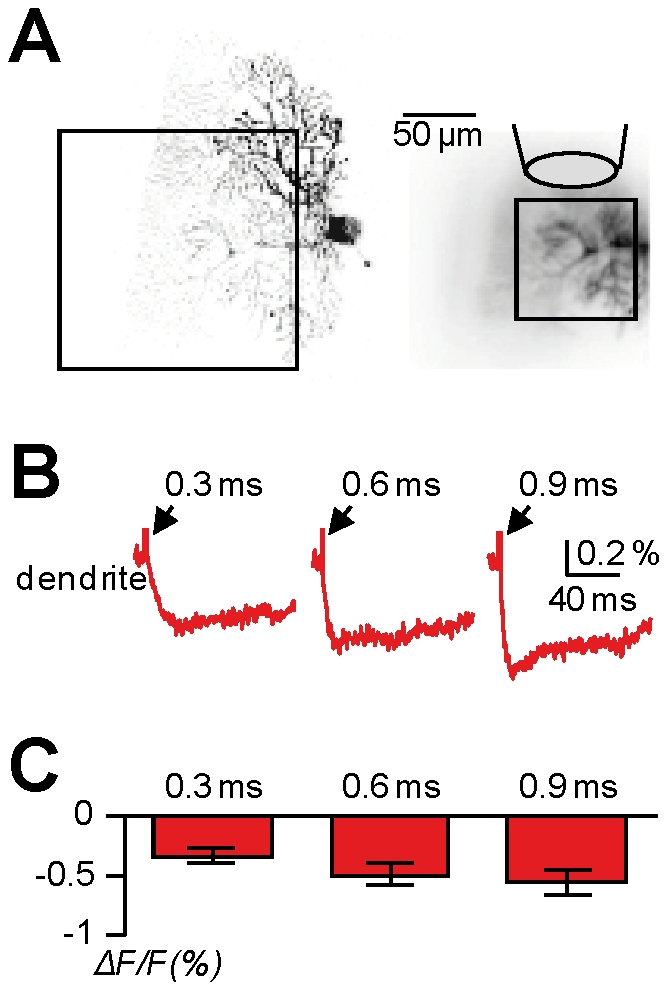
Polarity of dendritic fluorescence signal in response to wide-field GABA photorelease in PNs. **A.** (Left)- Image of a PN; the dendritic area in the recording position is outlined. (Right) Dendritic area of recording with a large ROI (∼80 µm×80 µm); caged-glutamate is perfused over the visualised area of the dendrite and wide-field illumination was used for photolysis (configuration of Fig. 1D). **B.** Membrane potential optical signals (red traces) associated with GABA photorelease by UV applications of 0.3 ms, 0.6 ms and 0.9 ms without patch recording; all traces are from averages of 4 trials. **C.** Mean ± SD (N = 5 cells) of the dendritic membrane potential optical signals associated with GABA photorelease by UV applications of 0.3 ms, 0.6 ms and 0.9 ms without patch recording.

Simultaneous voltage imaging and GABA photorelease is a powerful approach to spatially monitor GABA-mediated membrane potential changes and to investigate local phenomena of signal integration involving inhibitory signals. We have recently shown that membrane potential imaging without concomitant patch clamp recording allows measurements under physiological Cl^−^ homeostasis [17]. Because Cl^−^ mediates the response of GABA_A_ receptors and the intracellular Cl^−^ concentration varies during development [30] (and may not be uniform in a neuron [31]–[33]), membrane potential imaging is a unique tool to record GABAergic signals with physiological polarity and size. In a previous study [17], we have reported optical recordings of inhibitory synaptic potentials, elicited by electrical stimulation of presynaptic interneurons, in CA1 hippocampal pyramidal neurons. These experiments provided a measurement of physiological Cl^−^ mediated membrane potential changes from different neuronal sites. Nevertheless, because many hippocampal interneurons form multiple contacts onto different dendritic segments [34], the measured voltage signal could be in principle the integration of Cl^−^ currents occurring in different regions of the cell. In contrast, simultaneous voltage imaging and GABA photorelease allows recording of Cl^−^ mediated membrane potential changes from the same site where these signals are generated. Thus, we measured fluorescence changes elicited by GABA uncaging from locally perfused DPNI-GABA (configuration of Figure 1B), in different areas of CA1 hippocampal pyramidal neurons. In the cell of Figure 4A, we recorded from three different positions (outlined with different colours) while also changing the position of the perfusion electrode and focusing on a ROI near the beam of the localised perfusion. We performed measurements in the basal dendrites (red outline), in the apical dendritic segment proximal to the soma located in the stratum radiatum (blue outline), and in the distal apical dendrites located in the stratum lacunosum-moleculare (green outline). Negative changes of fluorescence, consistent with hyperpolarising membrane potential changes of a few millivolts, were recorded from all three positions (Figure 4B). In 5 cells tested (Figure 4C), the percentage change of fluorescence (mean±SD) was 0.17±0.03 in the basal dendrites, 0.31±0.12 in the proximal dendrites (stratum radiatum) and 0.28±0.11 in the distal dendrites (stratum lacunosum-moleculare). We performed the same type of experiment in PNs. In the cell of Figure 5A, we perfused DPNI-GABA using a large pipette and photo-released GABA over the whole visualised dendritic field (Figure 1D). By uncaging GABA with a variable UV pulse duration (0.3–0.9 ms) and at maximal UV intensity, we recorded negative dendritic changes of fluorescence varying from ∼0.35% to ∼0.6% (Figure 5B). In 5 cells tested (Figure 5C), the percentage changes of fluorescence (mean±SD), associated with GABA photorelease episodes of 0.3 ms, 0.6 ms and 0.9 ms of maximal UV intensity, were 0.33±0.16, 0.49±0.19 and 0.55±0.23 respectively. In summary, in both CA1 hippocampal pyramidal neurons and PNs during the fifth postnatal week and in all dendritic regions tested, the optical signals were consistent with a Cl^−^ reversal potential <−85 mV and an equivalent intracellular Cl^−^ concentration of 5 mM or less.

### Use of GABA photorelease to investigate shunting inhibition

**Figure 6:**
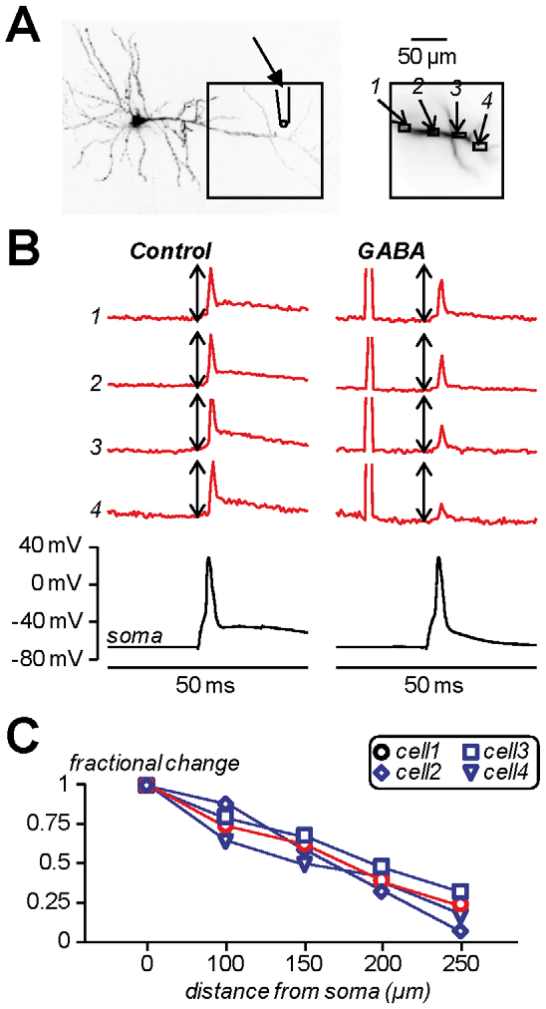
Investigating shunting inhibition using combined voltage imaging and local gaba photorelease. **A.** Image of a CA1 hippocampal pyramidal neuron; the dendritic area in the recording position is outlined and four ROIs (∼16 µm×8 µm) are shown on the right; caged-glutamate is applied locally in the position indicated (Figure 1B). **B.** Membrane potential optical signals from ROIs *1*–*4* (red traces) and somatic recordings (black traces) associated with one back-propagating action potential elicited by somatic current injection in control condition (left) and 15 ms after an episode of 1 ms GABA photorelease (right). The optical signals are normalised to the peaks of the spike under control conditions; the length of the double-arrow corresponds to the peak; all traces are from single trials. An animation of this experiment is available in Movie S1. **C.** Plot of the peak of the spike after GABA photorelease normalised to the peak of the spike under control conditions against the distance from the soma in the cell shown in A (red symbols) and in three other cells (blue symbols).

Propagation of action potentials in the axon or back-propagation of action potentials in the dendrites can be strongly regulated by local changes of membrane conductance due to opening of GABA_A_ receptors; a phenomenon known as “shunting inhibition” [35]. Simultaneous voltage imaging and GABA photorelease is an ideal approach to investigate shunting inhibition. In the CA1 hippocampal pyramidal neuron of Figure 6A, we explored the effect of local GABA photorelease on the back-propagating action potential in the apical dendrite. We applied DPNI-GABA using a patch pipette in the distal part of the dendrite at ∼200 µm from the soma using the configuration shown in Figure 1B. We then compared the fluorescence change associated with a back-propagating action potential under control conditions (Figure 6B, left traces) and 15 ms after an episode of GABA photorelease (Figure 6B, right traces) from four ROIs. The amplitude of the somatic action potential was the same in both cases, but local GABA uncaging decreased the size of the peak of the fluorescence change associated with the back-propagating action potential progressively with the distance from the soma. Although the size of the optical signal was not calibrated in terms of membrane potential change (in mV), the measurement of the fractional decrease of the action potential peak at each individual site was accurate because of the linear behaviour of the voltage fluorescence signal. An animation of this experiment is available in Movie S1. The effect of shunting inhibition on the back-propagating action potential was consistently observed in all 4 CA1 hippocampal pyramidal neurons tested using the same protocol (Figure 6C). It must be pointed out that shunting inhibition produced by GABA uncaging is different from physiological shunting inhibition, since the distribution of activated GABA receptors is different from that obtained by synaptic activation. Nevertheless, shunting inhibition produced by GABA photorelease is a model of physiological shunting inhibition permitting a separation of presynaptic and postsynaptic effects, a clear localisation of the activated GABA receptors and the possibility to perform pharmacological studies that cannot be done using presynaptic stimulation.

## Discussion

In this report we describe how to perform simultaneous voltage imaging and photolytic release of l-glutamate or GABA. These measurements were based on the resolution of several technical problems, including the modification of the microscope for dual illumination and the development and assessment of simple protocols for local perfusion of caged compounds and the regulation of the dimension of the photolysis spot. The caged compounds used in this report are stable, pharmacologically inert, efficient and kinetically fast [11], [14]. Thus, the approach described here can be used for a variety of applications. The optimal size of the area of photolysis will depend on the particular application. For some applications, the optimal diameter of the illumination spot is 1–5 µm. This can be achieved either by using a UV laser [4], [20], [22] or by two photon uncaging [7], [36]–[38]. UV lasers can be coupled to our microscope through the epifluorescence pathway replacing the UV LED that was used in the present configuration. For other applications a less localised photolysis or the uncaging over the whole field of view are more advantageous. This can be done, as described here, using UV LED illumination. In this report we have shown how to use simultaneous voltage imaging and photolysis as tools to calibrate the fluorescence signals in terms of absolute membrane potential changes. This is a notable result because it allows the possibility to quantitatively compare signals from different sites, a requirement necessary to analyse the spatial distribution of membrane potential changes. The method described here is valid in the cell types where the glutamate receptor conductance becomes dominant over the background conductance and can be possibly extended to other cell types using different caged compounds. We also showed how to record Cl^−^ mediated membrane potential changes under physiological conditions and how to investigate local mechanisms of signal integration such as shunting inhibition. The most salient finding from these experiments is the extreme focal restriction of the shunting effect and the resulting rapid loss in action potential backpropagation. This despite the fact that the GABA uncaging occurred over a relatively large area – considerably larger than what would be expected for a synaptic signal. These applications were used to validate the method, thus opening the gate to novel physiological investigations. The apparatus to achieve these measurements is based on commercially available equipment that can be adapted to conventional microscopes.

## Supporting Information

Movie S1 Animation of a shunting inhibition experiment using combined voltage imaging and local GABA photorelease. Back-propagating action in a CA1 hippocampal pyramidal neuron apical dendrite under control conditions and 15 ms after an episode of 1 ms GABA photorelease.
(WMV)
